# Using clinical practice guidelines to manage dengue: a qualitative study in a Malaysian hospital

**DOI:** 10.1186/s12879-019-3680-5

**Published:** 2019-01-11

**Authors:** Alexandra Wharton-Smith, Judith Green, Ee Chin Loh, Alexander Gorrie, Sharifah Faridah Syed Omar, Loraine Bacchus, Lucy Chai See Lum

**Affiliations:** 1 London School of Hygiene and Tropical Medicine, 15-17 Tavistock Place, London, WC1H 9SH UK; 2 School of Population Health & Environmental Sciences, King’s College London, London, SE1 1UL UK; 3 University of Malaya, 50603 Kuala Lumpur, Malaysia

**Keywords:** Dengue, Case management, Clinical practice guidelines, Malaysia, Qualitative

## Abstract

**Background:**

Malaysia has rising dengue incidence. World Health Organization clinical practice guidelines for managing dengue have been adapted by the Ministry of Health in Malaysia, with evidence of good awareness by clinicians. However, dengue mortality has not reduced. This study aimed to explore the challenges of dengue management for Medical Officers, with a particular focus on use of clinical practice guidelines.

**Methods:**

Qualitative study using six focus groups and 14 semi-structured interviews with doctors responsible for dengue management at a large tertiary hospital in Malaysia.

**Results:**

Dengue was recognised as difficult to diagnose and manage. Wide awareness and use of both WHO and Ministry of Health guidelines was reported, but several limitations noted in their coverage of particular patient groups. However, the phrase ‘guidelines’ also referred to local algorithms for fluid management, which were less clinically evidence-based. Where Medical Officers were well trained in the appropriate use of evidence-based guidelines, barriers to use included: the potential for ‘following the algorithm’ to undermine junior clinicians’ claims to clinical expertise; inability to recognise the pattern of clinical progress; and lack of clinical experience. Other reported barriers to improved case management were resource constraints, poor referral practices, and insufficient awareness of the need for timely help seeking.

**Conclusions:**

Awareness of clinical practice guidelines is a necessary, but not sufficient, condition for optimal dengue management. In high prevalence settings, all clinical staff would benefit from regular dengue management training which should include diagnosis, practice in monitoring disease progression and the use of clinical practice guidelines in a range of clinical contexts.

**Electronic supplementary material:**

The online version of this article (10.1186/s12879-019-3680-5) contains supplementary material, which is available to authorized users.

## Background

Dengue is estimated to pose a risk to half of the world’s population [1], with approximately 390 million infections per year globally [1]. There has been a 30-fold increase in global incidence over the last 50 years [2, 3] and the manifestation of more severe forms of dengue [2]. Dengue has been hyper-endemic for decades in south-east Asia, and the region has the highest incidence of dengue in the world [4]. In Malaysia, dengue is a major cause of morbidity and infectious disease mortality [5], and becoming more prevalent, with national annual incidence increasing from around 6543 reported cases in 1995 [6] to 100,028 cases and 231 deaths in 2016 [7]. A cross-sectional seroepidemiological study conducted in 2008 found that 91.6% of participants (*n* = 1000, aged 35–74 years old, male and female, of Malay, Chinese and Indian ethnicity, from different geographical locations) were dengue seropositive [8]. The authors suggested that the population’s high dengue IgG seropositivity implies that dengue is likely to remain endemic in Malaysia in the long-term [8], presenting a considerable burden of disease and economic costs [9].

Pang and Loh describe the escalating trends of hospitalisation and dengue deaths in Malaysia and suggest that “improper clinical management” in Malaysia is a cause for concern [10]. Key to improving dengue management is the better utilisation of evidence-based Clinical Practice Guidelines (CPGs). CPGs based on clinical trials data and expert consensus have been developed by the World Health Organization (WHO) [11] and adapted in Malaysia by the Ministry of Health [12]. For these CPGs to improve dengue management, clinicians need to be aware of them, trained in their use and willing to use them. CPGs also need to be routinely embedded in clinical practice, such that care can be co-ordinated across primary, secondary and tertiary levels and across departments within health care facilities. Quantitative survey evidence suggests a high degree of awareness and utilization of dengue management CPGs among Malaysian doctors, in both the public and private sectors (98 and 86% respectively) [13]. However, as in other settings, these self-reports of high utilization may not reflect actual practice. One case-note review found a wide range of adherence to current Malaysian guidelines depending on the type of facility. Low adherence (or poor recording) particularly in outpatient settings was reported [14], Malaysia is not alone in facing these challenges. Despite the dissemination and uptake of the WHO CPGs by ministries of health, several studies undertaken in dengue endemic countries have highlighted challenges, with observations of suboptimal compliance to the CPGs [10]. One study from Sri Lanka reported that fluid management for dengue patients at a tertiary health centre was “governed by consensus guidelines rather than by strong research evidence” [15]. Cheah et al. suggest a need for research that focuses on exploring the barriers to CPG implementation, particularly “in view of the large percentage of deaths due to fluid overload” [5]. There is, then, an urgent need to understand better the barriers to managing dengue in practice in high prevalence settings, and specifically to understand what would facilitate better utilisation of CPGs. This study aimed to look in detail at Medical Officers’ dengue case management at an urban hospital in Malaysia, using qualitative methods to identify barriers to CPG adherence, and facilitators of improved patient care.

## Methods

As our aim was to explore what happens in practice and why, rather than to gather reports of knowledge or adherence, a qualitative methodology was appropriate [16]. The study used focus groups and semi-structured interviews with doctors at a tertiary level, urban, public hospital in Malaysia to explore their knowledge and experience of managing dengue using CPGs, and their views of the wider challenges in dengue care pathways and case management. The hospital was selected as it receives high numbers of dengue cases, and hosts Medical Officers who have completed two years of post-qualification house officer training in a range of previous settings. This is followed by at least another year of clinical experience before joining this hospital’s post graduate training post, which leads to a specialist qualification upon completion of the four-year training. This cohort of Medical Officers thus provides a sample of doctors with a range of perspectives and experiences of managing dengue. Medical Officers were purposively sampled across the Emergency, Primary Care Clinic and Medical departments and stratified by their year of the post-graduate programme (1, 2, 3 or 4).

### Data collection

Focus group discussions (FGDs) are a productive method for accessing not just what people know, but (through interaction in the group) how that knowledge is shared, reproduced and perhaps challenged in practice [17]. To facilitate sharing of experiences among peers, FGDs were homogenous by year group [17]. Six FGDs were held, including 54 participants in total. An additional 14 Medical Officers were interviewed individually, to explore themes in more depth [18], and allow for disclosure of experiences that might be difficult to discuss in focus groups with peers. Semi-structured interviews enable flexibility, prompting for greater detail, and a greater rapport with the interviewer than is usual with more structured interviews. These are important when the aim is to generate more open answers, which elaborate on what is important to the participant, rather than simply eliciting opinions or responses to pre-determined questions. Topic guides for both FGDs and individual interviews included prompts for describing dengue case management, experience of difficult cases, training, diagnosis, management, clinician’s practice and patient demands (see Additional file 1). FGDs and interviews were conducted in English, audio recorded and transcribed verbatim, with any local language words used left un-translated in the transcripts, with an English translation in parentheses for context.

### Data analysis

The data were analysed using a modified thematic content analysis approach, using an initial coding frame based on the topic guide (deductive analysis), but with additional themes identified through close line-by-line interrogation/open coding using techniques from constant comparative methods (inductive analysis) [19]. Data collection was carried out during 2014–2016. Data extracts used in this paper are tagged with the data source (either Focus Group Discussion [FGD] or individual interview [I]) and the participant’s year group (1, 2, 3, or 4) of the Medical Officer programme. All identifying personal and hospital names have been anonymised to preserve confidentiality.

## Results

### Knowledge of dengue presentation and diagnosis

Dengue was very familiar to all participants, with most reporting having seen hundreds of dengue patients during their medical careers to date. They could describe how to take a history and conduct the required tests and examinations. This knowledge was recalled from training in medical school, from ‘on the ward’ experience as house officers, and also dedicated dengue workshops during clinical practice, both before their current posting and at the study hospital. In general, Medical Officers described learning how to manage dengue from their “*seniors*”, describing clinical training as “*It’s like ‘monkey see, monkey do.*’” *(FGD 4)*.

*“Once you become a Houseman, like when I was in [a hospital in another Malaysian city], we have our own dengue ward, so you have to know everything because I hear and hold everything as a Houseman. As a Houseman, I have dengue patients, I take blood myself, I see the patient.” (FGD 1).*

Dengue was widely noted as inherently difficult to diagnose, and to manage, and requiring considerable clinical expertise and experience, particularly for severe cases and atypical presentations:

*“I think it’s a bit harder when [patients] are still febrile, when it’s still early on the disease and, sometimes you do have some atypical pattern…I have seen a couple of atypical cases where it looks totally different and somehow somebody decided to send an NS1 antigen [test] and it turns out to be positive. So that, that case reminds me that if nobody sends the NS1, you probably would not have got it as a case of dengue.” (FGD 2).*

Many recalled similar experiences of ‘near misses’ in diagnosis. However, in general, the Year 4 Medical Officers were more willing to express uncertainty than those from other years. Given their relatively greater clinical experience, this may well reflect their own growing awareness of the potential complications of dengue progression:

*“I think severe [dengue] is a challenge for me. Usually I’m not confident enough, I have to admit that. Even though [I have] seen a lot of cases, but is usually quite mild – not as bad as here. When I’m at [another tertiary level hospital], when a patient went into decompensated shock, ICU will take over and we stop going there at all […]So here, especially during on call, let’s say resus [resuscitation], then in severe cases is a bit of challenge lah,* 1 *I have to be, I have to admit I was not confident actually. But after doing ID [an attachment to the infectious disease ward], I felt a bit more confident especially after going for the course, I learned a lot after that, and based on experience from colleagues as well.” (FGD 4).*

For the Year 4 FGD, greater experience of a wide range of patient presentations and clinical progressions had reinforced the need for careful prompts for relevant history at admission, and awareness of the inevitable uncertainties of disease progression in individual patients. This experience had, perhaps, made them both more confident in their own management decisions, but also aware of the complexities of dengue, meaning they could admit to their own uncertainty. Growing competence in diagnosis and management, and greater clinical autonomy, could mean conflict with less experienced clinical colleagues, as this Medical Officer describes:

*“[The patient] came in with only a day or two of fever. But we treated because we trusted our clinical instinct – we believe he has dengue. And we actually replaced [fluids] for him rather than treat him with antibiotics…apparently he had that history of some exposure to rats. So we have a bit of argument with our colleagues in another department, because they were saying that this was actually a septic shock rather than dengue shock.” (FGD 4).*

Across all years, many Medical Officers had experienced deaths of patients from dengue, which were particularly troubling. Beyond the emotional toll of the loss of any patient, there was a sense that as dengue deaths were preventable in theory, they therefore potentially reflected on the clinical team. The system of national investigations of all dengue deaths reinforced the sense that a death could reflect missed opportunities for improvement in case management.

*“My personal experience during my dengue death* ***,*** *initially I don’t have what she said lah, the guilt first…did I miss something? Is there something I could do more to prevent this death? Because my patient was a 40-year-old lady came in with dengue 3rd day [of fever]. Came in for blood check. Then subsequently admitted that night itself [and] decided to succumb in the midnight at 1 o’clock where no monitoring, nobody else is around. When they found her, her severe abdominal pain subsequently go into hypotensive shock and died* ***.*** *... Some disease we can try our best to prevent death, but not all the time we succeed* ***.”*** *(FGD 3).*

*“I mean you had a bad experience [of a dengue patient dying] it will be with you forever.” (FGD 3).*

### Familiarity with guidelines

Both the WHO guidelines and the Malaysian MOH adaptation, which is reproduced as a booklet, summarised in a leaflet, available in the hospital, with a flow chart to summarise management, were recognised by all Medical Officers. Year 1 and 2 Medical Officers expressed more familiarity with the WHO CPGs, whilst Year 3 and 4 Medical Officers reported using both the Malaysian MOH and WHO guidelines. The different guidelines were compared in terms of their “*physician friendly*” utility, with the MOH CPGs being described as *“A quick reference guide…[Laughter] Malaysia CPG. The 8 pages one, that is easier to read than the real CPG. There is a flow chart”* [20]. The consensus in one Year 3 FGD was that in other large urban hospitals, the Malaysian CPGs are strictly followed; all of these participants reported using them.

Year 4 Medical Officers had a range of perspectives on the usefulness of the algorithm for fluid management included in the CPGs; one said it was useful to picture the algorithm when managing dengue whilst a colleague said that understanding the pathophysiology of dengue was key to moving from an “*automatic kind of management”* to a ‘clinical’ approach and individualised care, based on the pathophysiology of dengue and specific patient factors:

*“Each time I see a severe dengue, in my mind I’ll just imagine the algorithm picture, try to manage from there actually. Go by clinical [presentation]…The decompensated dengue shock [picture] – the algorithm, step-by-step, I try to remember, recall that and try to manage the patient as that lah.” (FGD 4).*

However, in addition to the WHO and MOH CPGs, Medical Officers also recalled a range of what they called ‘local guidelines’. This generally referred to specific fluid regimes, which were adopted by particular hospitals as algorithms applied to all patients irrespective of their clinical signs. These were reported from Medical Officers across the data set, from a range of previous posts. These local guidelines were described as using a “lump sum” approach to fluid management. These could be based on estimates of a patient’s body weight:

*Participant 1: Unfortunately, last time was a bit more lump-sum lah. I agree that it has been a bit lump sum. Assuming that the body system is like that when this is the expected maintenance that you can take. So, rather than rigid calculation [...] we sort of like agak-agak* [estimate].

Moderator: OK, how to “*agak*”?

*Participant 1: Look at the patient’s size. It’s ranges from 4 to 6, [pints of fluid] usually, it depends on the patient. (FGD 4).*

Local guidelines were recalled as specifying ‘fixed’ recommended fluid regimes to be applied to all patients:

*“I think when in [a tertiary urban] hospital, almost everybody gets 10 at the first hour and then they rapidly cut down to 7 cc, 5cc and 3cc. Most of them we end up with 3cc, rarely 1 cc. And all the patients will get six hourly blood every single day. Regardless of how well they are. Routine. Six hourly routine.” (FGD 3).*

*“When I was at [another hospital] they have a regime back then. For example, if there’s a bit more [haemo]concentration, we tend to run fluids a bit more even though the patient is OK clinically. It’s like a fixed thing somehow, for example, female, the haematocrit more than 40, we give a bolus of fluid.” (FGD 4).*

These recollections of ‘fixed’ regimes were in contrast to what was reported as ‘clinical’ management at the study site, as well as being recommended in the Malaysian and WHO CPGs, whereby use of fluids was guided by the clinician’s assessment of the individual patient, carefully tailored to the patient’s vital signs and actual weight. This requires more frequent monitoring and assessments by the doctors, and thus greater workload. Thus, recognition of the limitations of local guidelines, with their ‘fixed’ protocols for fluid regimes, was only in retrospect: no Medical Officers reported feeling disquiet with these regimes at the time they used them.

The key clinical issue for dengue treatment is fluid management. However, tailoring fluid regimes to individual patients, taking account of (for example) co-morbidities or other complex health issues is not straightforward. Participants described their current practice as ranging from strict adherence to the fluid regime detailed in CPGs (in the form of an algorithm) to a “clinical” judgement call, reliant on clinical experience, expertise and patient factors.

### Using guidelines requires experience, yet potentially undercuts expertise

Guidelines have complex relationships with clinical experience, expertise and identity. At a pragmatic level, with regard to the WHO and MOH guidelines, participants discussed gaps between what they called the “theory” of how dengue should be managed, as codified in the CPGs, and actual case management in practice. First, there were limitations in the coverage of available guidelines, which did not detail requirements for many patient groups, including elderly patients, pregnant women, and patients with heart disease and/or renal failure. Such patients required the physician to “*be flexible*” (Year 2 Medical Officer); as “*we cannot really just follow the guidelines strictly, it’s [the guidelines are] more like a clinical base…presentation varies and the way you manage varies*.” *(FGD 4).*

Second, even with classic presentations, Medical Officers described the necessity of what they called *“a clinical approach”*, which required flexibility in interpreting guidelines for specific patients. A ‘clinical approach’ drew on *“instinct”*, individual judgement, and experience: embodied and esoteric skills which could not be codified. Thus, many stressed that adherence to the guidelines was inevitably shaped by clinical contingencies. In noting the importance of re-assessing the patient to determine the best individualised management, one participant commented: *“It’s just a guide lah. It’s a guide but you cannot be rigid to it…you cannot simply follow the guidelines.” (FGD 4).*

Given this concept of esoteric judgement as central to being a competent professional, the very idea of an algorithm for guiding management could be problematic, as it suggested the possibility of ‘blind following’, without using professional discretion. Rigid adherence is fundamentally opposed to clinical autonomy. That this is a possible understanding of the role of guidelines was evident in accounts from all year groups. This Medical Officer’s account, for instance, uses the positive term ‘clinical’ to describe an approach that is in contrast to that of what is here called following the *“step thing”*:

*“I find dengue, the general idea, understanding the pathophysiology behind the disease is very important. I think I knew all this previously. It’s just that things went a bit more automatic kind of management...Unfortunately, I’m not so [such a fan of the] algorithm, I’m a bit clinical. Although the basic concept of algorithm I understand, follow, but I can’t go with the step thing.” (FGD 4).*

For the earlier career Medical Officers in particular, whose clinical status was perhaps less secure, these kinds of implicit contrasts between simply ‘following’ algorithms and using professional discretion could mean that using guidelines has the potential at least to suggest inability to make professional, autonomous clinical judgements, or as simply unnecessary for those at ‘doctor level’ already. This Year 2 clinician, for instance, cites experiential learning, encapsulated in terms such as “rhythm”, “touch” and “judgement” as being fundamental to professional clinical practice, whereas ‘learning’ guidelines was something that could be done by anyone, with or without clinical experience:

*“What I feel, regardless of WHO or Malaysian CPG, what they are trying to [do] is just the same thing, I mean we are at doctor level already, we got the rhythm. I mean it’s just like A go to B, B go to C but more important is like whether you can pick it out when you see the patient, when you touch the patient…that’s more relevant rather than just go on theory…do this, do this…who cannot talk all these things? I mean, everybody go for exam, know what to write, but how [to do] the assessment, the judgement is very individual.” (FGD 2).*

Many of the more experienced clinicians described guidelines as only making sense in the light of clinical experience: as this improved, then the rationales for algorithms became clearer. Thus, learning guidelines was not the same as proficiency in using guidelines. Ability to put knowledge of guidelines into practice could be fostered by good clinical training, as this Medical Officer describes, reflecting on workshops they have attended which moved their practice from the ‘rigid’ approach to that of understanding the reasons for particular steps in the algorithm. It was this understanding which made it possible to use clinical discretion:

*“I think the idea is understanding the leakage and intravascular tissue part of it. I think everyone knows about it, it’s just that, maybe a bit clear after going for the courses and understanding a bit more, and the emphasis that was given. When you actually see the patient and then you are there, then you put in things into perspective, you find that your decisions are a bit more solid lah, like you understand why. Because it used to be, ‘I know leakage, I know about intravascular tissues,’ but then things were definitely more automatic last time, I mean standard 6 pints, no calculations. Last time was always like that. Until I came here then I realized that probably can go into other extreme [fluid overload]. But once you understand, I think, that’s what I felt. I guess the difference [is] I managed to apply pathophysiology rather than just know it – and you know, automatic. I guess the course was eye opener to sort of like make things more – how to say, there’s a reason lah, applicable reason to do it.” (FGD 4).*

To be effective, though, such training had to be with ‘real’ patients, particularly to provide exposure to severe and a range of atypical presentations:

*“…you need to see severe dengue, if you see enough of severe dengue, you manage yourself before then is fine, maybe if you want to pass, if you want to say you are confident in managing dengue. You must manage a certain amount of dengue patient, maybe 10 or 20, then you are okay.” (FGD 3).*

### Patient pathways: Reported barriers to effective management

Typically, dengue patients were reported to enter the hospital through the hospital’s own Primary Care Clinic, through the Emergency Department or to have transferred into the Medical Department from an external health facility. The patient would then be seen by a doctor who would conduct a clinical assessment, including a physical exam and history-taking and, when deemed appropriate, order blood tests, such as a full blood count (FBC) and a non-structural protein 1 (NS1) antigen rapid test for dengue.

Three major barriers to effective patient pathways into the Medical Department were identified by participants: lack of public understanding of dengue symptoms and warning signs, which could delay help seeking; poor diagnosis and management in primary care, which both delayed appropriate treatment and potentially obscured clinical signs; and poor referral and communication pathways within hospitals.

#### Public understanding

Delays in help-seeking were reported as resulting from poor public knowledge about dengue symptoms, and the warning signs that should be heeded when swift help-seeking was appropriate. Despite wide public awareness of dengue and its primary management through vector control, Medical Officers drew on their experiences of late presentations to suggest that patients still lacked education on the necessity for timely self-referral:

*“I think most people are still not very clear about it [warning signs]. They do know somewhat that you need to drink water and things like that, but a lot them are still very unclear ...we generally don’t have a very good system of educating the public about what is important.” (FGD 1).*

*“The community lah. Seldom have correct information. […] Advertisement only tells them what’s dengue and all that. But they didn’t write what happen later, what you should do, only say seek medical attention.” (FGD 3).*

*“See if they come in early, they know what to look out for, hopefully we can prevent, hopefully the mortality reduces.” (FGD 1).*

A second problem was the use of non-biomedical therapies. A range of folk medicines were reportedly used by patients to manage dengue fever, including ‘rhino’ water (a traditional Chinese drink used for its perceived “cooling” effect), papaya leaves, which were believed to cure dengue, and advertised as efficacious, crab soup and porcupine Bezoar stone. Such remedies were seen as problematic by the Medical Officers, not just because there was no evidence of their effectiveness, but because they could delay timely help-seeking, or worsen symptoms, resulting in more serious presentation:

*“You know… because usually, they buy the water, the rhinoceros brand water which contains acid. I have one patient; she drank … 6 bottles. At the end patient’s haematocrit dropped. Then kidney not clean. Then nothing improving. So [she was] admitted… [and] transferred to ICU.” (I 3).*

*“Patients have this tendency to always listen to their neighbours, daun betik [papaya leaves] lah, don’t know what. And then they come too late, right? I am not against it actually but I wish they would stop from seeing other people. Just see a doctor if you are not well, you know.” (FGD 1).*

#### Primary care management

Primary care doctors, across all sectors, were widely reported as inadequately managing dengue in the community, particularly through offering inappropriate therapies such as antibiotics or NSAIDs. These practices persisted, it was argued, because primary care doctors had no source of feedback on their prior management decisions, and no continuing professional development to modernise their diagnostic or therapeutic knowledge:

*“They are from the old school you know, everything is IM [intramuscular] jabs, steroids and all that, that takes some moving, things like dengue, they don’t know, they just refer and that’s about it. Because we don’t have that system where they can see what happened to the patient, but it would be very nice if we have got more time or if we got personnel in charge, they can liaise back with the GP so that they don’t make the same mistake again. You don’t give IM jabs, you don’t give antibiotics unnecessarily, that’s when all the [antimicrobial] resistance is coming lah.” (FGD 1).*

*“I think the... first doctor regardless the public setting, the private setting. If the patient comes in with the fever, and we do short check, don’t ignore it. Don’t just give him the chart and ‘eh go home read’. Must explain, I think not many doctors do that, they don’t understand what is actually the danger signs of having dengue, they just look at the platelets then that’s it for patient.” (FGD 3).*

The inappropriate prescription of antibiotics for dengue patients prior to their arrival at the hospital was reported as a particularly challenging issue. This could delay appropriate treatment seeking by patients, but could also skew clinical presentation and laboratory test results, specifically, of nausea, diarrhoea and liver derangement.

*“[One of the challenges is] late presentation of dengue…because they are happy with the antibiotics so they stay back at home not presenting to the hospital or main centres with their symptoms.” (I 7).*

*“…because they patients will go back [from the GP] and think ‘oh you know I’m taking this already, I’m fine, I’ll be safe from dengue’ and they will not come in [to hospital] till they are really, really ill and bleeding away…” (I 4).*

#### Inter-department coordination

Colleagues within the hospital were also reported as creating challenges in achieving good care pathways. Across the FGDs and interviews, incomplete history taking and incorrect diagnoses by ‘other departments’, primarily those responsible for front line assessment, were flagged as problematic. Front line colleagues were reported as failing to provide necessary information when making intra-hospital referrals:

*“...some of the doctors… tend to be dealing direct questions just to save time, like a checklist…kind of like narrow thoughts, just focus on one diagnosis which may be misleading instead of following up to consider seven different diagnoses which will be proven by the investigation.” (FGD 1).*

*“Very often I have to go and retake the history.” (FGD 1).*

Within the hospital, participants stressed an aim of “shared ownership” for dengue patient management, to achieve the goal of accountability and care for patients across departments and between doctors and nurses. This was disrupted by what was referred to as *“cuci tangan”,* meaning colleagues in referring departments who want to “wash their hands” of a patient. ‘Washing hands’ implied not taking responsibility, by referring patients before taking a full history or recording accurate fluid intake, or waiting for another department to approve a discharge. “Hand washing” was a common complaint in all year groups about (other) admitting departments, if no one admitted to doing it themselves. There were a number of reasons suggested for “hand washing” within the hospital. First, it was attributed to lack of confidence in dengue management in other departments, often because clinicians lacked experience in dealing with severe presentations of dengue, and were unwilling to take the risks of getting it wrong:

*“What I think is that, those departments like ED department, and then the primary care department, there is really a lack of experience in handling all the dengue cases. So that’s why if they need more exposure like attachment with the ID ward.” (FGD 1).*

*“I think the first line… for the ED… they need to know what is dengue. If you think you can discharge, be confident and discharge. Don’t wait for us and when to upgrade from trauma to [ICU]… or discharge…Don’t just ‘cuci tangan’ [wash hands of it].” (FGD 3).*

Others suggested that resource constraints placed pressure on busy clinicians to make rushed judgements.

*“I think it just boils down to manpower, MOs [Medical Officers]…We need more people who know how to manage dengue.” (FGD 3).*

Nursing staff were a crucial link for effective communication about patients. Well-informed nurses who are well-trained in dengue management were described as a vital source of the necessary detail on vital signs and fluid intake, and as a crucial part of the clinical ward team, taking responsibility for initiating assessment or therapeutic interventions:

*Participant 1: “Meaning that in a few cases that, when they think that the patient is not well, they will inform us. Like tachycardia, if patients is like not eating, or not passing urine. They will inform [us]. 3 o’clock, 2 o’clock. That is excellent.”*

*Participant 2: “Actually Ward X nurses are very well trained. They pick up the narrowing. They might be still well, but they will say, ‘Doctor, BP ini dah tak cantik [this BP doesn’t seem well], narrow.’ So, at least they alert you early. Sometimes they even check the glycaemic and BP on their own, because they think the patient is giddy.”*

*Participant 1: “…they will suggest us to reduce the drip. They will phone us: ‘the patient was on like 5cc for a long time, dah lamalah, doctor nak keluarkan kah patient? Patient dah boleh tidur, kencing’ [It’s been a while. Doctor do you want to take out? Patient can now sleep and pass urine]. [There is] ownership.”*

*Participant 4: “The nurse says ‘patient looks a bit…heart rate was low, although she was still fever at that point, I think she is ready for medicine, so can you please come.’ The nurses that actually picked up the sign said we could actually intervene early, so we started a drip.” (FGD 3).*

In contrast, where nurses were perceived to be less well trained, there was little sense of shared ownership, and lack of access to vital information about patients’ deteriorations could be a barrier to good management:

*“I think… let’s say the support staff [nurses] need to be much more well trained than we already have because it is there- sometimes they don’t pick up the signs that patient is already deteriorating because most of the time we are not there to assess.” (FGD 3).*

Senior staff availability to advise when necessary was also seen as vital for achieving effective shared ownership and thus good patient management: if seniors could be relied on to help when necessary, more junior clinical staff could be confident of taking responsibility, and perhaps less likely to “wash their hands” of complex cases:

*Participant 1: “Usually dengue, ‘I need you to come down’, they will drop everything and come. Usually they can come within half an hour.”*

*Participant 2: “They will cause it’s dengue…so it’s understood that we need them immediately.” (FGD 3).*

*“Unfortunately… I think dengue is such a big thing in Malaysia, I think the chain of responsibility has to go up. Because we can’t take that responsibility although we know to do, we still can’t take the responsibility, through experiences, I think this has happened many times, if you don’t inform, you’ll get questioned why you never inform. Things like that… Seems like our emphasis is there, although patient might be fine but then you get things like “oh, you manage alone?” It’s the chain of thing… It’s the way that is working here, so it’s the local practice. So we just follow…” (FGD 4).*

## Discussion

This study has added to the small literature on dengue case management in practice, and is the first qualitative study of the role of clinical practice guidelines in dengue management in Malaysia. We have identified good awareness of WHO and Malaysian CPGs among Medical Officers in a large tertiary hospital in Malaysia, but also widespread reports of ‘local’ guidelines, that generally advocated fixed regimes of fluid management. In settings with very high dengue admission rates and resource constraints, these may well be used to streamline patient throughput. They may be problematic, however, if patients requiring more tailored fluid regimes are not identified. Given previous survey evidence of good awareness and utilization of CPGs [13], this is concerning, as many participants reported having been unaware of how to calculate accurate fluid management. The reported high awareness of, and utilization of, ‘guidelines’ in surveys [13] may well refer to these fixed regime algorithms for fluid management.

Further, we have shown that knowledge and availability of guidelines is a necessary, but not sufficient, condition of evidence based dengue management. As studies of many other conditions and settings, from the UK to Tanzania, have found [21–23], knowledge of guidelines in theory does not always reflect clinical practice. Reported use of clinical guidelines may be a poor guide to actual use. Decision aids such as guidelines have to be embedded in routine patient management, rather than simply available, in order to impact on patient care.

As participants in this study noted, there are some pragmatic barriers to current guidelines, including lack of detail on particular patient groups. More broadly though, clinical practice guidelines have complex relationships with important components of professional practice such as discretion, experiential learning and autonomy. One tension in the use of guidelines is that they require considerable clinical expertise to use appropriately, yet the use of a formal protocol also (at a symbolic level) can undermine claims to clinical expertise, particularly when those claims are precarious, as they can be for more junior clinicians. The accounts of Medical Officers in this study illustrate these inherent challenges of embedding CPGs in practice. That is, they are most helpful when there is a body of professional experience to draw upon. For the earlier career Medical Officers in particular, adherence to guidelines can sit uneasily with the need to demonstrate professional competence. The findings from participants in this study suggest that utilization of CPGs to improve dengue management will require considerable investment in continuing training of clinicians (including nursing staff) not only in infectious disease wards, but also in referring departments such as emergency departments and in primary care. Importantly, providing guidelines in clinical settings is only one component of the multi-dimensional approaches needed for improved dengue case management. This study has reiterated the need for all community and hospital settings involved in dengue management to receive regularly updated training in the appropriate use of those guidelines in a range of clinical scenarios.

Finally, we comment on the participants’ views of the context of CPG uptake, particularly the wider barriers arising from referral delays. Attributing treatment delays to public ignorance and poor practice in primary care is perhaps a universal claim of hospital physicians, and Medical Officers’ reports of patient and GP practices which contribute to treatment delays cannot be taken as evidence of those practices. Indeed, a study of dengue mortality in Malaysia found little evidence that these delays were a factor [24]. However, in a context of changing epidemiology of dengue in Malaysia, with higher incidence in adults, there may well be scope for improving the primary/secondary care referral pathways, and looking in more detail at what does happen in primary care. The medical training of primary care physicians, in the private sector in particular, could be augmented by a formal requirement for continuing professional development in general, especially updating training on dengue case management. Similarly, attributions of public ignorance about dengue warning signs and the need for help-seeking as contributing to dengue mortality may well be more likely to be indicators of clinicians’ frustration with late presentations rather than necessarily barriers. However, as widespread use of folk remedies for dengue has been reported, together with beliefs that biomedicine cannot offer a ‘cure’ [25], there may be scope for exploring how to improve public understanding of the role of biomedical management, the need for recognising dengue symptoms and the importance of timely help-seeking.

### Strengths and limitations

This study included a relatively small number of Medical Officers, from one hospital; the findings may not be generalisable to other settings. However, our participants represented clinicians with experiences from hospitals across Malaysia, who were currently at a tertiary centre: their knowledge and practice is likely to be broadly representative of national good practice. As we were reliant on Medical Officers’ reports of previous practice (such as adopting ‘fixed’ fluid regimes) and current practice (such as inadequate information in intra-hospital referrals), we had no access to data which would shed light on what did happen in other settings, or what the reasons might be for particular practices. Ethnographic methods would be needed to explore whether, for instance, fixed regimes reflected unmanageably high workloads in urban hospitals, as the CPG recommends fluid management based on regular and frequent clinical assessment of patients. Further research to explore these issues in more detail would be useful.

## Conclusions

In a large Malaysian hospital, we found a number of barriers to optimum care pathways for dengue patients. These results shed some light on possible reasons for continuing dengue mortality in the region. Despite identifying good awareness of clinical practice guidelines, our study highlighted the need for guidance of Medical Officers by senior clinicians who have been trained and are experienced in dengue case management. Our findings also suggest caution in optimism about uptake of WHO and Malaysian MOH guidelines as the only approach to improving dengue case management. First, although knowledge and awareness was high, there were also widespread reports of local adaptations that included ‘fixed’ regimes for fluid management. Second, knowledge and awareness are necessary, but not sufficient, conditions for the use of CPGs. Training in guideline utilisation, based on the range of clinical scenarios likely to be encountered in practice is needed. Further qualitative research is needed to explore how CPGs are (or are not) routinely embedded in practice in dengue endemic regions.

## Additional file

Additional file 1: CPGs to manage dengue_topic guide, interview and focus group discussion topic guide (DOCX 19 kb)

## Footnotes

1 “Lah” is a colloquial Malaysian term that provides emphasis to the preceding statement
