# Supplementary material for: Using clinical practice guidelines to manage dengue: a qualitative study in a Malaysian hospital
Source: BMC Infect Dis. 2019 Jan 11;19:45. doi: 10.1186/s12879-019-3680-5 (PMC6329084; doi:10.1186/s12879-019-3680-5)
Supplement: Supplementary file 1 — CPGs to manage dengue_topic guide, interview and focus group discussion topic guide (DOCX 19 kb) [file 12879_2019_3680_MOESM1_ESM.docx]

**Interview and Focus Group Discussion Topic Guide**

**Dengue presentation**

1. Which are the common dengue clinical features that you see?

**Dengue training/teaching**

1. How did you learn about dengue?

2. Which reading materials did you access?

3. Any guidelines?

4. How did you learn to clinically manage dengue?

5. What do you think about the learning method?

6. What is your experience of dengue training at other hospitals?

7. What would you like to learn more about related to dengue?

**Dengue patients**

1.What is the usual scenario you encounter with dengue patients?

2. How do you think we are doing here (in managing dengue cases)?

**Management**

1. How about when you admit the patient, what do you think of patient monitoring?

1. What can you tell me about the fluid regime?

3. What do you think about the kind of support you receive to manage dengue cases?

**Dengue deaths**

1. Have you encountered any dengue deaths?
2. What is the commonest cause of dengue death?
3. How could dengue deaths in hospitals be avoided?

**Patient expectations**

1. What are the patients’ expectations for care when they have dengue?

**5. Recommendations**

1. Thinking generally about the management and discharge processes, how can the quality of care for dengue patients be improved?

2. Do you have any other recommendations on this topic or anything else that you would like to add?

3. Is there anyone else you suggest that I speak to?

1. Do you have any questions for me?

**Thank you so much for your time.**
